# Methodology for Exploring Patterns of Epigenetic Information in Cancer Cells Using Data Mining Technique

**DOI:** 10.3390/healthcare9121652

**Published:** 2021-11-29

**Authors:** Hanan Aljuaid, Hanan A. Hosni Mahmoud

**Affiliations:** 1 Department of Computer Sciences, College of Computer and Information Sciences, Princess Nourah Bint Abdulrahman University, Riyadh 11047, Saudi Arabia; haaljuaid@pnu.edu.sa; 2 Department of Computer and Systems Engineering, Faculty of Engineering, University of Alexandria, Alexandria 21544, Egypt

**Keywords:** epigenetic pattern, genes, cancer groups, data mining, clustering

## Abstract

Epigenetic changes are a necessary characteristic of all cancer types. Tumor cells usually target genetic changes and epigenetic alterations as well. It is most beneficial to identify epigenetic similar features among cancer various types to be able to discover the appropriate treatments. The existence of epigenetic alteration profiles can aid in targeting this goal. In this paper, we propose a new technique applying data mining and clustering methodologies for cancer epigenetic changes analysis. The proposed technique aims to detect common patterns of epigenetic changes in various cancer types. We demonstrated the validation of the new technique by detecting epigenetic patterns across seven cancer types and by determining epigenetic similarities among various cancer types. The experimental results demonstrate that common epigenetic patterns do exist across these cancer types. Additionally, epigenetic gene analysis performed on the associated genes found a strong relationship with the development of various types of cancer and proved high risk across the studied cancer types. We utilized the frequent pattern data mining approach to represent cancer types compactly in the promoters for some epigenetic marks. Utilizing the built frequent pattern item set, the most frequent items are identified and yield the group of the bi-clusters of these patterns. Experimental results of the proposed method are shown to have a success rate of 88% in detecting cancer types according to specific epigenetic pattern.

## 1. Introduction

We would like to introduce a complete understanding of the genomic changes in cancer cells through data mining techniques. The idea of alterations of the cellular genome of cancer is very crucial. Our paper will focus on developing novel and efficient techniques to compare DNA sequences of normal cells with those of cancerous cells. We intend to develop techniques for detecting epigenetic and genomic changes in cancer. We utilize computational biology to create useful techniques to classify cancerous cells according to genome alteration. Detecting epigenetic changes in cancer is not a trivial issue. Those changes cannot be detected in a lab because of the large amount of data derived from variations in the mutations in genes caused by cancer and also because of the many kinds of cancer. Bioinformatics can apply tools to extract and detect such mutations and establish association rules that associate specific epigenetic changes and specific kinds of cancer.

Cancer research is one of the major research areas in the medical field. Cancer identification has always been clinical-based. The cancer identification methods have many limitations [1,2,3]. Tumor classification is heterogeneous and molecularly different [4]. Systematic approaches that are based on gene expression analysis have been introduced [5]. Microarray technology has permitted the simultaneous monitoring of genes, which stirred recent development in cancer identification utilizing gene expression information [6]. Different identification methods from data mining techniques have been utilized to cancer classification. Gene expression information is different data that data mining had previously dispensed. The genome has high dimensionality. It contains tens of thousands of genes. Additionally, many genes are unrelated to cancer identification. It is clear that existing taxonomy methods are not intended to handle this kind of data efficiently [7].

Gene selection can be performed prior to cancer grouping. Gene selection aids in reducing data size. Gene selection also eliminates a large number of unrelated genes and increases classification accuracy. These are several issues that are related to the biological context of this research. These issues are the statistical significance versus biological significance of cancer classifiers. For example, asymmetrical classification problems and gene contamination problems. It is essential to study both points and their linked issues [8,9,10].

In this research, we investigated a three-way clustering data mining technique; the 3WC is an exhaustive cancer types and epigenetics pair analysis. The method 3WC utilizes a three-way clustering technique to discover the coherent epigenetic patterns within various cancers. We utilized the 3WC technique to explore six acute epigenetic patterns in relation to seven cancer groups and recognized a substantial relationship between cancer and epigenetic patterns. The results expose the existence of a dependable epigenetic alteration inclination within these cancers. 

Past research on computerized cancerous cell identification focus on computerized classification of cancerous cells using genome alteration. The authors in [11] proposed a mass investigation scheme through the fuzzy C-means technique using genome alteration patterns that are supervised by deep-CNN (CNN is a convolutional neural network) to classify the cancer cells. In [12], the authors devised a block region segmentation in a collective process to partition the tumor into the cell digital maps. Authors in [13] brought together a watershed technique that performed a granular splitting of the cancerous cells in the cell map with genome identification by merging epigenetic regions with the same average values. In [14], the authors proposed an algorithm to detect cell epigenetic masses in the cell map and devised an aggressive segmentation procedure. In [15], the authors employed the AlexNet CNN to detect epigenetic masses in cell map images of the various cancer types.

In the phase of cells classification, the authors in [16] produced a Bayesian network technique to establish some genome features that are introduced to program diagnosis of cancer groups. In [17], the authors introduced a decision tree classifier for epigenetic pattern extraction of cancer masses, and they compared it to an SVM (support vector machines) classifier. The authors in [18] employed a deep-CNN to calculate the probability of the existence of cancerous cells by training the CNN with a dataset in the gene paradigm. In [19], they used deep learning methods to predict cancerous cell existence by training data of 420 epigenetic maps. In [20], the authors built a feature-extraction CNN for benign mass identification using a decision tree. The research in [21] emphasized a neural network techniques to identify cancerous cells genome map and further classify them. In [22], the authors offered a cell map pattern analysis using machine learning to detect a patient’s risk of specific cancer types. In [23], the authors projected a minimum support analysis process using the second and third moment for pattern extraction in a breast cancer cell map. The authors in [24] introduced a CAD (computer aided design) mechanism of genetic pattern extraction to identify common features through forest classification.

This paper is divided into the following sections: Section 2 discusses the methodology and the utilized dataset. Section 3 describes the experimental results. Section 4 depicts the discussion of the results, while conclusions are summarized in Section 4.

## 2. Materials and Methods

In the following subsections, we are describing the dataset used and the methodology of the three-way clustering technique.

### 2.1. Data Set

We studied the epigenetic similarities across various cancer types of seven cancer types, including adenocarcinoma human alveolar basal epithelial cells (AHC), human erythroleukemic cell line (HEC), liver hepatocellular carcinoma (LHC), human colorectal adenocarcinoma cell line with epithelial morphology (HCC), clonal derivative tumors (CDT), multiple myeloma cancer (MMC), and Burkitt lymphoma cancer (BLC). We excluded the epigenetic patterns that are not found in these cancer types and utilized only the patterns that characterize these cancer types demonstrated in Table 1. The data are included in the NIH Epigenome Dataset [4].

### 2.2. Methodology

**Figure 1:**
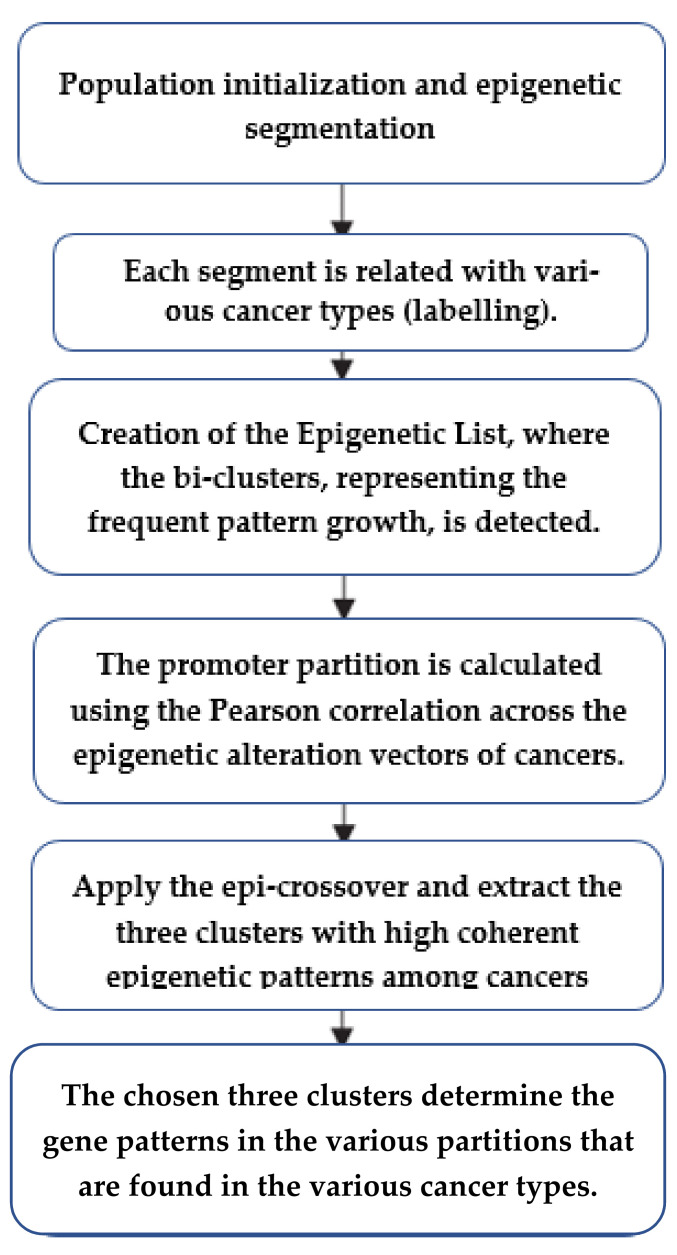
The flowchart of the proposed three-way clustering technique (3WC) includes preprocessing of the epigenetic marks of various cancer types and detecting the three clusters with coherent epigenetic marks among various cancer types.

We are proposing a three-way clustering technique (3WC) to observe the epigenetic pattern of various types of cancer. The proposed technique identifies the combinatorial various epigenetic pattern in gene parts. The technique also observes similar epigenetic patterns among various cancer types. The introduced 3WC technique has several phases, as depicted in the three phases described below. The first phase is a preprocessing stage that modifies epigenetic patterns data in various cancer types. The second phase detects the bi-clusters of the frequent pattern growth algorithm. The third phase coherently detects the three clusters with epigenetic alterations patterns among the various cancer types. The method is depicted in Figure 1.

The three phases are described as follows:

Phase 1: Population initialization is the phase where executing the preprocessing of the data of the epigenetic alterations of various cancers takes place. The epigenetic is segmented into segments. For each alteration map, we calculate the alteration count of every partition. Each segment is then related with the epigenetic alterations intensities for various cancer types. To observe the resultant noise from high tag counts in the experiments, the number of each alteration are normalized by count of the input utilizing linear transformation [25]. At the end, the distribution of the genome data in the various partitions is computed.

The preprocessing step observed six reads of seven groups in the promoter partitions. Assume Genome = {n1, n2,…, n7 } is representing the genes, Type = {p1, p2,…, p7} is representing the cancer groups, and Epig = {i1, i2,…, i7} represents the patterns. For all patterns, the profiles of various cancer groups in the partitions are presented in a matrix Dk = Type × Genome, where the rows represent the seven cancer types, and the columns represent the genes. Additionally, each column is a one column array corresponding to the gene profile of the promoter partition of gene j.

**Figure 2:**
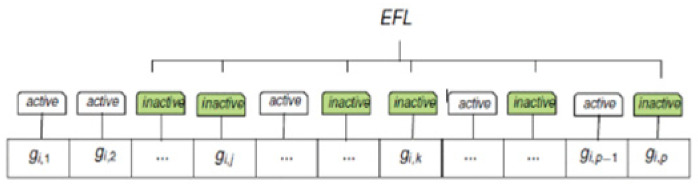
Epigenetic list of the patterns H3K9m3 in a usual tri-cluster with the same pattern in human erythroleukemic cell line (HEC) cancer.

Phase 2. Creation of the epigenetic list, where we detect the bi-clusters representing the frequent pattern growth of the epigenetic patterns. Succeeding the preprocessing of the epigenetic alteration matrix of the pattern, we calculate the correlation of the gene profiles for the bi-related cancers at the corresponding partition, and this results in a correlation matrix. The promoter partition is calculated using the Pearson correlation across the epigenetic alteration vectors of various cancer types. If the computed coefficient is greater than a specific threshold, the epigenetic trend in these cancer types is considered to be highly coherent. Then, we consider this cancer to be the same item set, which includes the various cancer types having the same epigenetic patterns. The threshold is set as 0.65 based on extensive experimentation for the epigenetic patterns to be highly coherent. Each epigenetic alteration is built using similar item sets for all promoters. The resultant item set determines the most important coherent patterns using the frequent pattern growth methodology [26]. Frequent pattern growth methodology is a mining technique that is developed for frequent pattern mining. In this paper, we utilized the frequent pattern tree algorithm to model cancers with the same gene patterns in a compact way in the promoters. The pattern tree mines the frequent patterns and excludes the unrelated data. A frequent patterns defines a set of cancer types that have the same epigenetic patterns in the most voted promoters. To identify the most important epigenetic patterns, we settled the support of genes to a minimum of 10% of the studied genes. In this phase, we identify the chosen gene set inversely and determine a gain of the bi-cluster. The bi-cluster is defined as a set of two things, namely genome and cancer. This identifies the cancers that possess the same epigenetic patterns (EFL) in the corresponding genes as depicted in Figure 2.

Phase 3. In this phase, we apply epi-crossover and extract the three clusters with high coherent epigenetic patterns among various cancers. After extracting the bi-clusters for the epigenetic pattern, we extract the three clusters and, naming the best value of the various gene patterns, we extract the three clusters. We calculate the bi-clusters intersection of each two epigenetic patterns, which are defined with the epigenetic patterns to get all the three clusters. This phase filters them with support less than the required minimum support. We iterate the procedure with the other epigenetic pattern until we analyze all the epigenetic patterns. We experimented with all the paths and chose only the optimal three clusters. The three clusters are defined as genes, cancer types, and the gene patterns with the same trend of gene patterns in various cancers. The chosen three clusters determine the gene patterns in the various partitions that are found in the various cancer types.

## 3. Experimental Results of the Genome Partitions

In our simulation, we utilized parallel MATLAB with CUDA tools that run Simulink in parallel. This software has the advantage of utilizing the computing resources by setting preferences. It has a complete processing multicore CPUs. We utilized a Parallel Computing Toolbox in MATLAB 4.1 on MATLAB Parallel Server (MPS).

From the pre-defined clusters, we can identify the required sets coupled with some significant gene patterns. To study the properties of these partitions, we implemented an ontology and pathway analysis through the bioinformatics named DAVID [4]. The important enrichment sets are determined with probability value < 0.006.

**Figure 3:**
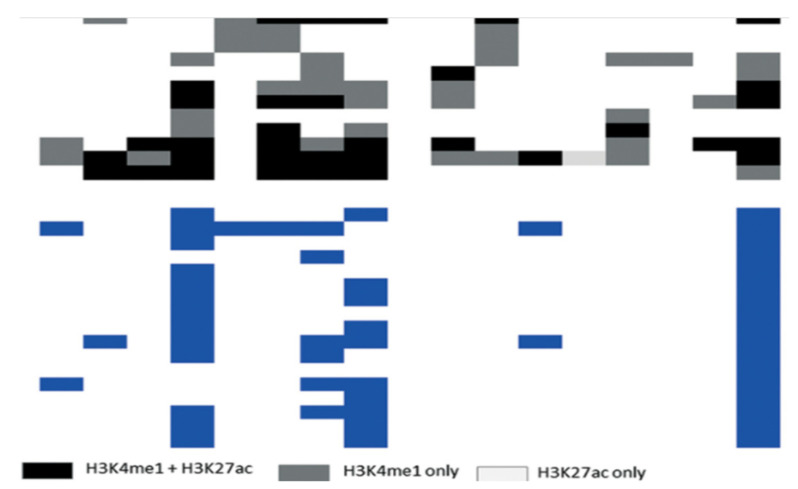
The epigenetic patterns H3K4me3 in a cancer groups CDT, LHC, HEC, and AHC.

We proposed a three-way clustering approach, 3WC, to identify the same epigenetic patterns among various cancers. 3WC was utilized on the genome epigenetic alteration maps of the chosen cancer types, namely: AHC, HEC, LHC, HCC, CDT, MMC, and BLC. For all epigenetic patterns, 3WC groups the promoters based on the definition of the epigenetic profiles along various cancer types. Figure 3 depicts bi-clusters of epigenetic pattern H3K9m1, which include excess genes with the same alteration pattern in four cancers, including AHC, CDT, LHC, and HEC. We conclude that the gene marks are the same in predetermined cancers. The gene patterns that are described by promoters in several cancer types are defined as epigenetic patterns, while various cancers can share the same patterns. This succeeded in significant results, with defining the H3K9m3/H3K9m2 and H3K36m3/H3K36m2, which are usually identified in breast cancer and lung carcinoma. The defined bi-clusters of these studied epigenetic patterns determine the two cancers (LHC and HCC) clustered with each other and have a large count of epigenetic patterns. This implies that they possess more similar epigenetic patterning.

**Figure 4:**
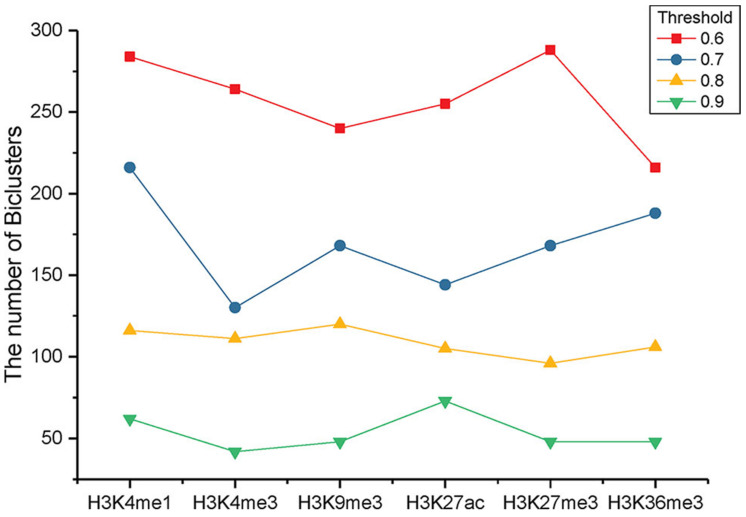
Experimental results of the bi-clusters thresholds versus various gene patterns. The comparison specifies that the correspondences of the epigenetic patterns are quite different. H3K4me3 has a small number of bi-clusters, while H3K4me1 and H3K27me3 have similar patterns across various cancer types.

To define the important alteration patterns, we reduce the minimal support to 10% of the studied genes. Utilization of diverse correlation thresholds can result in gaining various counts of bi-clusters for epigenetic patterns H3K9m1, H3K4m3, H3K9m3, H3K27m3, H3K36m3, and H3K27c, among these cancers, as depicted in Figure 4. The comparison clarifies that the similarity measures of these epigenetic patterns are various. Using various thresholds, the epigenetic pattern H3K4m3 is shown to have a small count of bi-clusters. This results in profiling with the least conserved variable patterns within these cancers than the other epigenetic patterns. On the opposite side, there are better similarity in the epigenetic patterns of H3K4m1 and H3K27m3 in the other various cancers. The quality of the epigenome is highly dependent on many environmental factors. This concludes that epigenome types can aid in the development of human cancers and diseases. The minimal threshold affects the trend in the various epigenetic patterns, so we define the bi-clusters as having a threshold of 0.72.

We can conclude that clear variations in the examined gene alterations are found. To determine the defined states and test the same patterns of the gene alterations, we utilized the grouping of these patterns using the defined clusters. For the calculation of the clusters’ intersections from various patterns, we used the three partitions with support greater than the predefined minimum support. The determined three clusters are grouped as triplets namely: genome partitions, cancers, and epigenetic patterns. The three clusters define that the promoters of these genomes show the same epigenetic alteration patterns in the cancers.

In Table 2, we are comparing our results from the three-way clustering mining technique with the ground truth found in the labeled data set in [4]. We concluded our results in the following confusion matrices for the epigenetic sets: H3K9m1, H3K4m3, H3K9m3, H3K27c, and H3K27m3 in the cancer groups CDT, LHC, MMC, and BLC.

## 4. Discussion

Utilization of epigenetic biomarkers at the bedside is a major breakthrough in cancer diagnosis and immersion of new drugs. Our proposed technique can be an aid for clinical practice as it introduces a cost-effective accurate relationship between epigenetic patterns and specific cancer types.

The experimental results conclude that each genomic group partition has the same combinatorial patterns across specific cancers. For instance, the epigenetic alterations (H3K4m3, H3K9m3, H3K27m3, and H3K36m3) are the same in large count of genes in the cancer groups AHC, LHC, and HEC. On the opposite side, few epigenetic patterns are significant in specific cancer groups. Among these investigated clusters, we find that the same patterns of H3kK27m3, H3K36m3, and H3K27c are found in small cancer groups, namely LHC and BLC. We have to clarify that these detected three clusters obviously reveal greater knowledge of the genes within these cancer groups.

**Figure 5:**
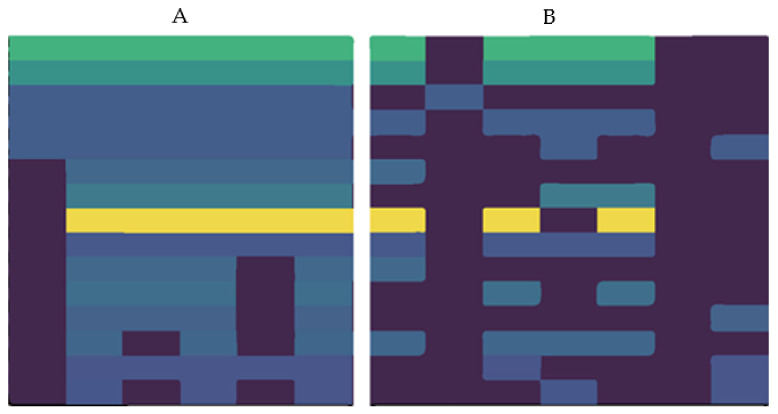
Patterns of the three clusters (epigenetic tri-clusters). (**A**) The marks are represented by columns, where rows represent clusters. (**B**) Columns represent cancer types, while rows represent clusters. Fold ratio is computed as the ratio of the genes count to the clusters in the genes.

Applying the 3WC algorithm to the genome in the dataset, we initially found 180 important three clusters. Figure 5 depicts the information of 16 coherent clusters with their epigenetic patterns, the cancers, and the achieved supports.

The simulation results specify that exact genomic sections share combinatorial patterns among various cancer types. For instance, the varying epigenetic pattern (H3kK27m3, H3K36m3, and H3K27c) is common in enormous genes in cancer types AHC, LHC, and HEC. Additionally, some epigenetic patterns are coherent in some cancer types across few clusters, namely LHC and BLC.

It was found that the specific gene sets exhibit coherent patterns in the specific cancer groups. Past research has found that the difference properties are far more distinct among high-function promoters than low-expression promoters, which implies that the color properties have a far more important effect on gene pattern regulation [27]. To study the specific properties of those specific genes in the various pathways, we executed an appropriate experiment using the DAVID technique [4]. We concluded that the related gene sets are mostly clear in the studied three clusters. We summarized the important living processes that they participate in.

As a conclusion, we detect that those gene groups were abundant in the three clusters and are enriched in the cancer-related properties. Table 1 enumerates the important GO properties of the three clusters with (probability-value <0.006). In the specified three cluster, the genes possess coherent patterns on the epigenetic: H3K9m1, H3K4m3, H3K9m3, H3K27c, and H3K27m3 in the cancer groups CDT, LHC, MMC, and BLC. In Table 1, we established the terms positive and negative regulations of cell proliferation of the apoptotic properties to indicate enriched gene groups. The experimental results depict that the detected genes in the three clusters are apparent for the cell apoptotic process. Additionally, we found that the positive regulation is also apparent in the gene group, which implies that these gene groups have a significant role in the regulation of these cancers.

The limitation of the proposed technique, the three clusters mining 3WC, is the high cost of the operation because we search the whole search space. Our future work will focus on developing statistical techniques to prune an efficient search space, to enhance the efficiency of discovering the three clusters.

## 5. Conclusions

In this paper, we introduced a three-way clustering technique 3WC for cancer gene study. We utilized the frequent pattern data mining approach to represent cancer types compactly in the promoters for some epigenetic marks. Utilizing the built frequent pattern item set, the most frequent items are identified and yield the group of the bi-clusters of these patterns. This proves that the same gene pattern in these cancers is within these genomic partitions. 3WC also uses the data mining technique to mine the specified three clusters using the bi-clusters of the studied epigenetic marks, detecting the combinatorial states in the genomic states and the corresponding epigenetic changes. We utilized the 3WC technique to discover the similar epigenetic patterns within the various cancer types.

The proposed method detects common patterns of epigenetic marks in various cancer types (epigenetic patterns across seven cancer types) and by finding epigenetic resemblances among cancer types. The experimental results found that mutual epigenetic patterns exist in these cancer types, with a strong relationship with various types of cancer, and found high risk across the studied cancer types. We built a frequent pattern item set and identified the group of the bi-clusters of these patterns. Experiments results of the proposed method achieved an accuracy of 88% in detecting cancer types according to a specific input epigenetic pattern.

We compared our results from the three way clustering mining technique with a labelled data set, and we validated our results for the epigenetic sets H3K9m1, H3K4m3, H3K9m3, H3K27c, and H3K27m3 in the cancer groups CDT, LHC, MMC, and BLC.

## Figures and Tables

**Table 1:** Marks that characterize the investigated cancer types.

H3K4m1	Is an epigenetic DNA alteration to the Histone protein H1
H3K4m3	Is an epigenetic DNA alteration to the Histone protein H3
H3K9m3	Is a tri-methylation at the 9th residue of the protein H3
H3K27c	Is the acetylation of the residue at terminal distance 27
H3K27m3	Is the tri-methylation of H3 protein
H3K36m3	Is the tri-methylation at the 36th position of the H3 protein

**Table 2:** Experimental results as related to ground truth.

Case	Age	Cancer Type	Diagnosis by 3WC	Diagnosis by Physician	Genome Detected by the Proposed 3WC	Genome Detected by Physician (Ground Truth)	Status
1	35	CDT	Epigenetic DNA alteration to the Histone protein H1	Epigenetic DNA alteration to the Histone protein H1	H3K9m1	H3K9m1	Match
2	61	LHC	Epigenetic DNA alteration to the Histone protein H3	Epigenetic DNA alteration to the Histone protein H3	H3K4m3	H3K4m3	Match
3	45	BLC	Epigenetic DNA alteration to the Histone protein H27	Epigenetic DNA alteration to the Histone protein H27	H3K27c	H3K27c	Match
4	32	BLC	Epigenetic DNA alteration to the Histone protein H3	Epigenetic DNA alteration to the Histone protein H1	H3K9m1	H3K9m3	Miss Match
5	25	CDT	Epigenetic DNA alteration to the Histone protein H1	Epigenetic DNA alteration to the Histone protein H1	H3K9m1	H3K9m1	Match
6	15	MMC	Epigenetic DNA alteration to the Histone protein H3	Epigenetic DNA alteration to the Histone protein H3	H3K27m3	H3K27m3	Match
7	67	CDT	Epigenetic DNA alteration to the Histone protein H3	Epigenetic DNA alteration to the Histone protein H1	H3K9m1	H3K9m1	Match in epigenetic, mismatch in diagnosis
8	75	LHC	Epigenetic DNA alteration to the Histone protein H27	Epigenetic DNA alteration to the Histone protein H3	H3K4m3	H3K4m3	Match in epigenetic, mismatch in diagnosis
9	32	MMC	Epigenetic DNA alteration to the Histone protein H27	Epigenetic DNA alteration to the Histone protein H27	H3K27c	H3K27c	Match
10	25	CDT	Epigenetic DNA alteration to the Histone protein H27	Epigenetic DNA alteration to the Histone protein H27	H3K27c	H3K27c	Match
11	52	LHC	Epigenetic DNA alteration to the Histone protein H1	Epigenetic DNA alteration to the Histone protein H1	H3K9m1	H3K9m1	Match
12	25	LHC	Epigenetic DNA alteration to the Histone protein H3	Epigenetic DNA alteration to the Histone protein H3	H3K4m3	H3K4m3	Match
13	5	MMC	Epigenetic DNA alteration to the Histone protein H3	Epigenetic DNA alteration to the Histone protein H3	H3K4m3	H3K4m3	Match
14	4	LHC	Epigenetic DNA alteration to the Histone protein m3	Epigenetic DNA alteration to the Histone protein m3	H3K27m3	H3K27m3	Match
15	6	MMC	Epigenetic DNA alteration to the Histone protein m3	Epigenetic DNA alteration to the Histone protein m3	H3K27m3	H3K27m3	Match
16	4.5	CDT	Epigenetic DNA alteration to the Histone protein m3	Epigenetic DNA alteration to the Histone protein m3	H3K27m3	H3K27m3	Match
17	3	LHC	Epigenetic DNA alteration to the Histone protein H3	Epigenetic DNA alteration to the Histone protein H3	H3K9m1	H3K9m3	Match
18	2.5	MMC	Epigenetic DNA alteration to the Histone protein H1	Epigenetic DNA alteration to the Histone protein H1	H3K9m1	H3K9m1	Match
19	5	CDT	Epigenetic DNA alteration to the Histone protein H3	Epigenetic DNA alteration to the Histone protein H1	H3K9m1	H3K9m3	Miss Match
20	25	LHC	Epigenetic DNA alteration to the Histone protein H1	Epigenetic DNA alteration to the Histone protein H1	H3K9m1	H3K9m1	Match
21	50	MMC	Epigenetic DNA alteration to the Histone protein H27	Epigenetic DNA alteration to the Histone protein H3	H3K4m3	H3Kc27	Miss Match
22	61	CDT	Epigenetic DNA alteration to the Histone protein H1	Epigenetic DNA alteration to the Histone protein H1	H3K9m1	H3K9m1	Match

## Data Availability

The data presented in this study are available on request from the corresponding author.
